# Effect of Cross-Linking on the Mechanical and Thermal Properties of Poly(amidoamine) Dendrimer/Poly(vinyl alcohol) Hybrid Membranes for CO_2_ Separation

**DOI:** 10.3390/membranes4020200

**Published:** 2014-04-08

**Authors:** Shuhong Duan, Teruhiko Kai, Takashi Saito, Kota Yamazaki, Kenichi Ikeda

**Affiliations:** Research Institute of Innovative Technology for the Earth (RITE), 9-2 Kizugawadai, Kizugawa-shi, Kyoto 619-0292, Japan; E-Mails: kai.te@rite.or.jp (T.K.); saitakashi@rite.or.jp (T.S.); yamazaki@rite.or.jp (K.Y.); kenikeda@rite.or.jp (K.I.)

**Keywords:** poly(amidoamine) dendrimer, poly(vinyl alcohol), cross-linker, CO_2_ separation, polymeric membrane

## Abstract

Poly(amidoamine) (PAMAM) dendrimers were incorporated into cross-linked poly(vinyl alcohol) (PVA) matrix to improve carbon dioxide (CO_2_) separation performance at elevated pressures. In our previous studies, PAMAM/PVA hybrid membranes showed high CO_2_ separation properties from CO_2_/H_2_ mixed gases. In this study, three types of organic Ti metal compounds were selected as PVA cross-linkers that were used to prepare PAMAM/cross-linked PVA hybrid membranes. Characterization of the PAMAM/cross-linked PVA hybrid membranes was conducted using nanoindentation and thermogravimetric analyses. The effects of the cross-linker and CO_2_ partial pressure in the feed gas on CO_2_ separation performance were discussed. H_2_O and CO_2_ sorption of the PAMAM/PVA hybrid membranes were investigated to explain the obtained CO_2_ separation efficiencies.

## 1. Introduction

Carbon dioxide (CO_2_) capture and storage (CCS) is generally considered as an option for climate change mitigation [1]. For practical application of the CCS technology, cost-effective methods for CO_2_ capture are required [2]. Many studies have focused on the development of effective CO_2_ capture and separation technologies [3], with membrane separation being one of the promising solutions because of its energy efficiency and operation simplicity. Membrane separation affords CO_2_ separation from a pressurized gas stream, for example through the integrated coal gasification combined cycle (IGCC) process. The high-pressure difference between the feed and permeate sides of the membrane provides sufficient driving force to conduct membrane separation in the absence of additional compressors and vacuum pumps, thus affording reduced CO_2_ separation costs [4].

Sirkar *et al.* [5,6,7] reported that a poly(amidoamine) (PAMAM) dendrimer-immobilized liquid membrane (ILM) can achieve a high CO_2_/N_2_ selectivity under atmospheric pressure. However, the dendrimer ILM had insufficient pressure tolerance for practical use because of the flow nature of the PAMAM dendrimer at or above room temperature. To improve CO_2_ separation performance at pressure difference conditions, hybrid membranes have been developed by incorporating or immobilizing a PAMAM dendrimer into a polymer matrix in our research group. The immobilization of the dendrimer in a cross-linked chitosan was successfully achieved by an *in situ* modification method, and the resulting membrane system demonstrated remarkable enhancement in CO_2_/N_2_ separation [8,9,10]. However, the limited loading of a PAMAM dendrimer (up to 30 wt %) and complex fabrication procedure have prompted for an alternative immobilization process. Recently, effective incorporation of a PAMAM dendrimer into a cross-linked poly(ethylene glycol) (PEG) was realized by photopolymerization of PEG dimethacrylate [11,12,13], and the resulting membrane showed a high CO_2_/H_2_ separation performance. Poly(vinyl alcohol) (PVA) has been used as a matrix to facilitate CO_2_ transport through the membrane because of its good compatibility with both the mobile and stationary carriers, high hydrophilicity, and good film-forming ability. Ho *et al.* [14,15] reported cross-linked poly(vinyl alcohol) membranes containing polyallylamine, amino acid salt, and potassium hydroxide, and were claimed to contain both mobile and stationary carriers. Matsuyama *et al.* [16] reported a polyethylenimine/poly(vinyl alcohol) (PEI/PVA) blend membrane with stationary carriers. Cai *et al.* [17] reported a PAAm/PVA blend membrane with selectivities of 80 and 58 for CO_2_ over N_2_ and CO_2_ over CH_4_, respectively. In our previous studies, a novel hybrid membrane of PAMAM dendrimer/cross-linked PVA was developed [18,19,20]. A PVA network was formed with a Ti-based cross-linker in the presence of a PAMAM dendrimer in aqueous media. PAMAM was successfully immobilized in a cross-linked PVA matrix. In this paper, three types of organic Ti metal compounds were selected as PVA cross-linkers that were used to prepare corresponding PAMAM/cross-linked PVA hybrid membranes. Characterization of the PAMAM/cross-linked PVA hybrid membranes was conducted using nanoindentation tolerance tests and thermogravimetric analysis (TGA). The influence of the type of cross-linker and CO_2_ partial pressure in the feed gas on CO_2_ separation performance was investigated. H_2_O and CO_2_ sorption of the PAMAM/PVA hybrid membranes were examined to determine the effect of the cross-linker on the CO_2_ separation properties.

## 2. Experimental Section

### 2.1. Materials

**Figure 1:**
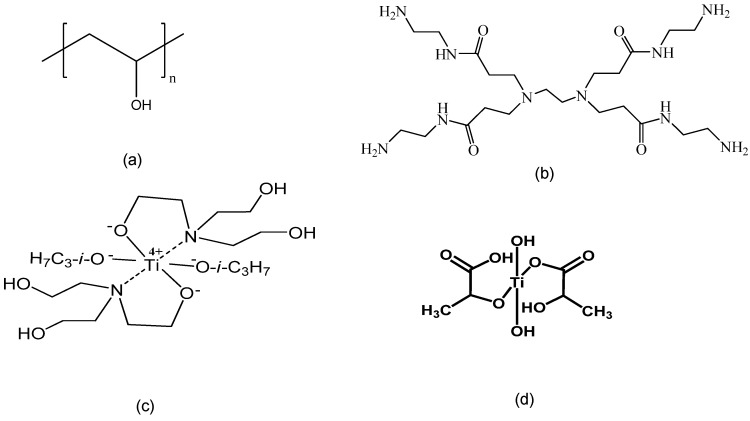
Chemical structures of PVA, PAMAM dendrimer, and Ti cross-linkers. (**a**) Poly(vinyl alcohol) (PVA) (*n* = ~2000, M_W_: 88,000); (**b**) Polyamidoamine (PAMAM) dendrimer (*G* = 0, M_W_: 516.7); (**c**) TC-400 cross-linker (di-isopropoxy-bis(triethanol aminato)titanium, M_W_: 462.4); (**d**) TC-310 cross-linker (di-hydroxy-bis(hydroxypropionic acid)titanate, M_W_: 260.0).

PVA (98+ mol % hydrolyzed; degree of polymerization, *n*: 2000) was purchased from Wako Pure Chemical (Osaka, Japan), three types of Ti-based cross-linkers (TC-400, TC-300, and T-2678) were obtained as 80 wt % isopropanol solutions from Matsumoto Fine Chemical (Chiba, Japan), and a PAMAM dendrimer (zero-generation) was purchased as a 20 wt % methanol solution from Sigma-Aldrich (St. Louis, MO, USA). The chemical structures of the materials mentioned above are shown in Figure 1. T-2678 cross-linker is not given chemical structure in detail. Other chemicals used were of analytical grade and used as received.

### 2.2. Membrane Preparation

PAMAM/PVA hybrid membranes (free-standing films) were prepared by casting a solution of PAMAM, PVA, and Ti cross-linker, at different weights, on a Teflon dish (inner diameter: 6.0 cm), as described in the reported study [6], followed by drying at room temperature for 2 days to evaporate the water.

**Figure 2:**
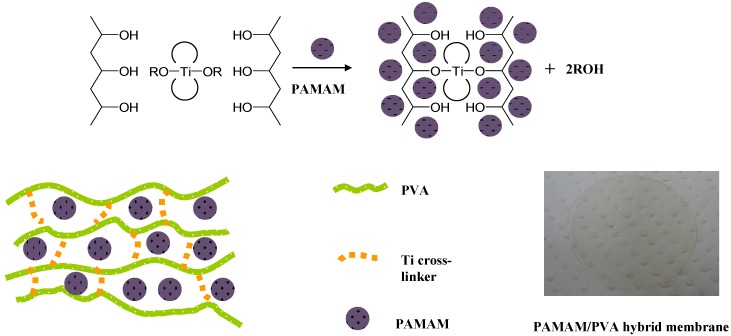
Schematic diagram of a PAMAM-immobilized Ti cross-linked PVA matrix.

Figure 2 shows a schematic diagram of the cross-linking process. PVA cross-linking was carried out with a Ti cross-linker in the presence of a PAMAM dendrimer in aqueous solution. The dendrimer was incorporated into the cross-linked PVA matrix to form a self-standing membrane. The representative resulting PAMAM/PVA hybrid membrane (PAMAM/PVA/TC-400 = 63.3/32.6/4.1 wt %) was transparent as seen in Figure 2.

To enhance gas permeance, thin composite membranes were prepared by casting the precursor solution of hybrid membrane onto a polyvinylidene difluoride (PVDF) support membrane. The PVDF porous support membrane (hydrophobic surface, average pore size of 0.1 µm, thickness of 120 µm, and porosity of 80%) was purchased from Millipore Com (Tokyo, Japan). The cast membrane was then dried overnight and heated at 120 °C for 10 min.

### 2.3. Permeation Experiments

The schematic diagram of the gas separation experiment setup used herein was provided in reference [8]. A CO_2_/H_2_ (80/20 v/v) gas mixture was humidified at 80% relative humidity (RH) and then fed into a flat-sheet membrane cell at a flow rate of 100 mL/min. As reported in our previous studies, our membranes require humidity levels as high as 80% RH to achieve a high separation performance. The CO_2_ partial pressures at the feed side were 80 and 560 kPa. Dry He was supplied at a flow rate of 10 mL/min to the permeate side of the cell as a sweep gas. The operating temperature was set at 40 °C. CO_2_ and H_2_ concentrations in both the feed and permeate gas were measured by gas chromatography. The permeance, *Q*, and selectivity, *α*_CO2/H2_, were calculated as expressed in references [8,9].

### 2.4. Membrane Characterization

Nanoindentation measurements of the composite membrane were performed on a Triboindenter TI-950 (Hysitron Inc., Minneapolis, MN, USA) using a Berkovich indenter (three-sided pyramidal), a quasistatic mode, and an indentation depth of ~200 nm.

TGA was carried out using a PerkinElmer Pyris 1 TGA system (PerkinELmer Japan Co., Ltd., Yokohama, Japan). Measurements were made by heating the samples from 30 °C to 300 °C under nitrogen atmosphere at a heating rate of 5 °C/min.

H_2_O sorption was performed on a BELSORP-aqua3 (BEL Japan, Inc., Osaka, Japan) at 40 °C. CO_2_ sorption was conducted on a BELSORP-BG (BEL Japan, Inc., Osaka, Japan) at 40 °C, 80% RH, and a pressure range of 0–800 kPa; Gas 1 was H_2_O and Gas 2 was CO_2_.

## 3. Results and Discussion

### 3.1. Nanoindentation Analysis

**Figure 3:**
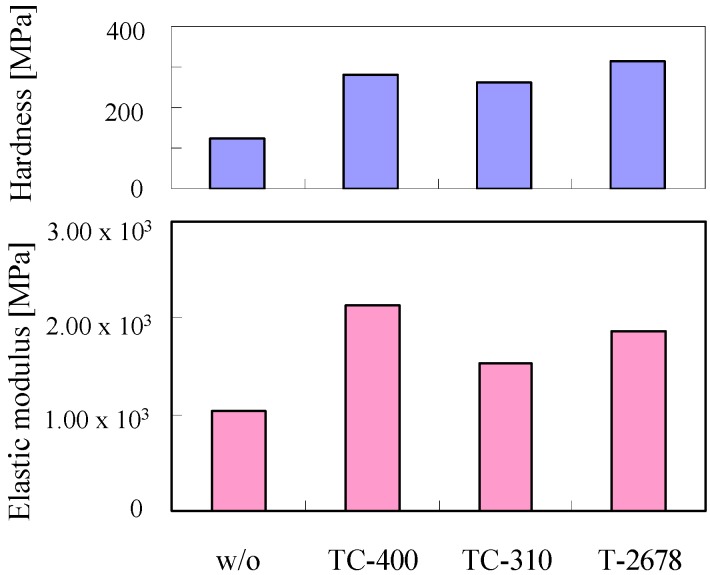
Nanoindentation tolerance evaluation of the hybrid membranes prepared in the absence (w/o) and presence of a cross-linking agent (TC-400, TC-310, or TC-2678).

Figure 3 shows the nanoindentation tolerance evaluation of the membranes prepared in the absence (w/o) and presence of a cross-linking agent. PAMAM/PVA/TV-400 hybrid membrane had the highest elastic modulus. PAMAM/PVA/TC-310 hybrid membrane displayed the highest hardness. PAMAM/PVA/T-2678 hybrid membrane had the lowest elastic modulus and hardness. However, the PAMAM/PVA hybrid membranes prepared in the presence of a cross-linking agent exhibited higher elastic modulus and hardness than those prepared in the absence of a cross-linking agent. The nanoindentation measurements suggest that cross-linking was effective to improve the mechanical strength of the PAMAM/PVA hybrid membranes.

### 3.2. TG Analysis

**Figure 4:**
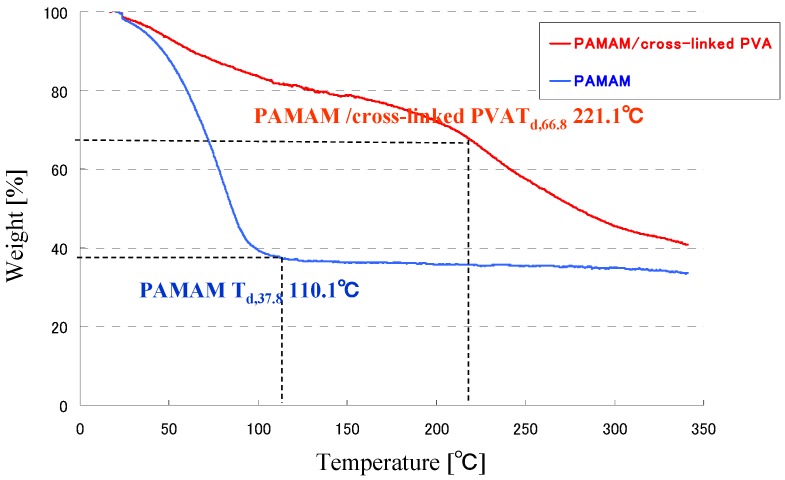
Thermogravimetric analysis (TGA) evaluation of the hybrid membrane and PAMAM dendrimer. TGA curves of (**a**) the PAMAM dendrimer; and (**b**) the PAMAM/cross-linked PVA hybrid membrane.

Thermal gravimetric analysis curves of the PAMAM dendrimer and PAMAM/PVA hybrid membrane (PAMAM/PVA/TC-400 = 63.3/32.6/4.1 wt %) are shown in Figure 4. The thermograph of PAMAM shows a mass loss of ~62.2% at 110 °C. The PAMAM/PVA hybrid membrane displays mass losses of ~17.6% at 110 °C and ~33.2% at 221 °C. The PAMAM-immobilized cross-linked PVA matrix shows considerably enhanced thermal stability when compared with the pure PAMAM dendrimer. The TG analysis suggests that cross-linking was effective to improve the thermal stability of the PAMAM/PVA hybrid membranes.

### 3.3. Effect of Cross-Linker on CO_2_ Separation Properties

**Figure 5:**
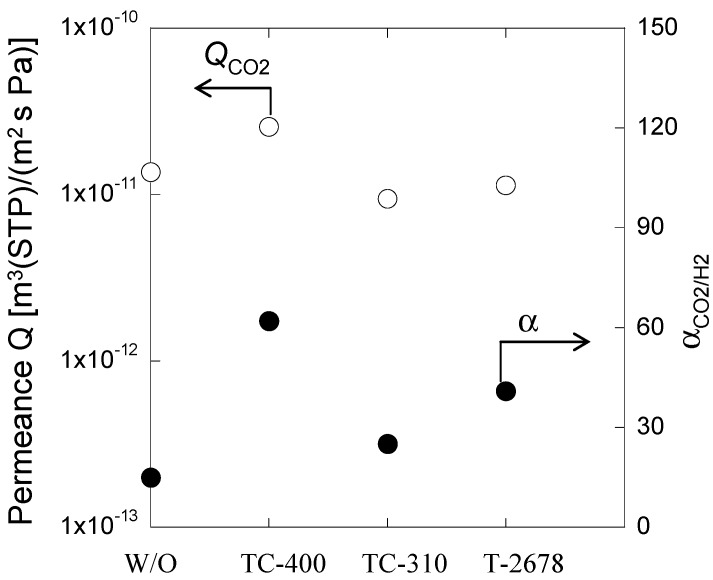
Effect of cross-linker on CO_2_ separation properties of PAMAM/cross-linked PVA membranes (thickness: 50 µm) at 80 kPa CO_2_ partial pressure and 80% relative humidity (RH).

CO_2_ gas separation properties of the PAMAM/PVA hybrid membranes at 40 °C and 80% RH are shown in Figure 5. The PAMAM/PVA hybrid membrane prepared in the absence of a cross-linker had a relatively low α_CO2/H2 _(CO_2_/H_2 _gas selectivity). Conversely, the PAMAM/PVA/TC-400 hybrid membrane showed the highest CO_2_ permeance (*Q*_CO2_) and α_CO2/H2_. The PAMAM/PVA hybrid membranes prepared in the presence of cross-linking agents TC-310 and T-2678 had lower *Q*_CO2_ values when compared with that of membranes prepared in the absence of a cross-linking agent; however, the former hybrid membranes displayed higher α_CO2/H2_ values. Three types of cross-linkers were used to improve the mechanical strength and thermal stability of the PAMAM/PVA hybrid membranes. Furthermore, although the cross-linking structure did not improve CO_2_ permeance (e.g., PAMAM/PVA/TC-310 and PAMAM/PVA/T-2678 hybrid membranes. Figure 5), the cross-linking structure efficiently reduced H_2_ permeance, thus resulting in improved CO_2_/H_2_ selectivity. Regarding the PAMAM/PVA/TC-400 hybrid membrane, the cross-linking structure was more effective in improving CO_2_ permeance, owing to the presence of the ethanol amino groups in TC-400 that would promote CO_2_ sorption.

**Figure 6:**
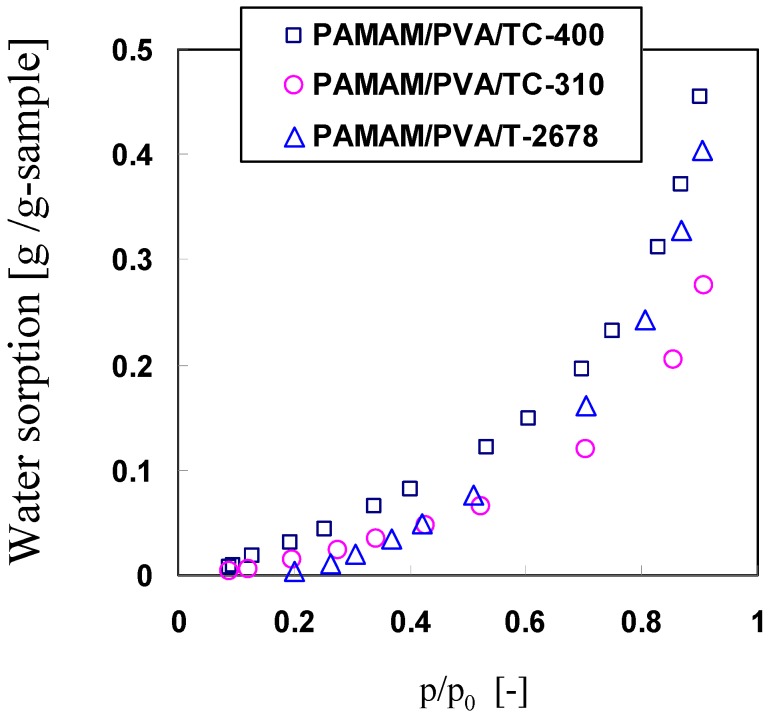
Effect of cross-linker on water sorption properties of the PAMAM/cross-linked PVA membranes at 40 °C.

**Figure 7:**
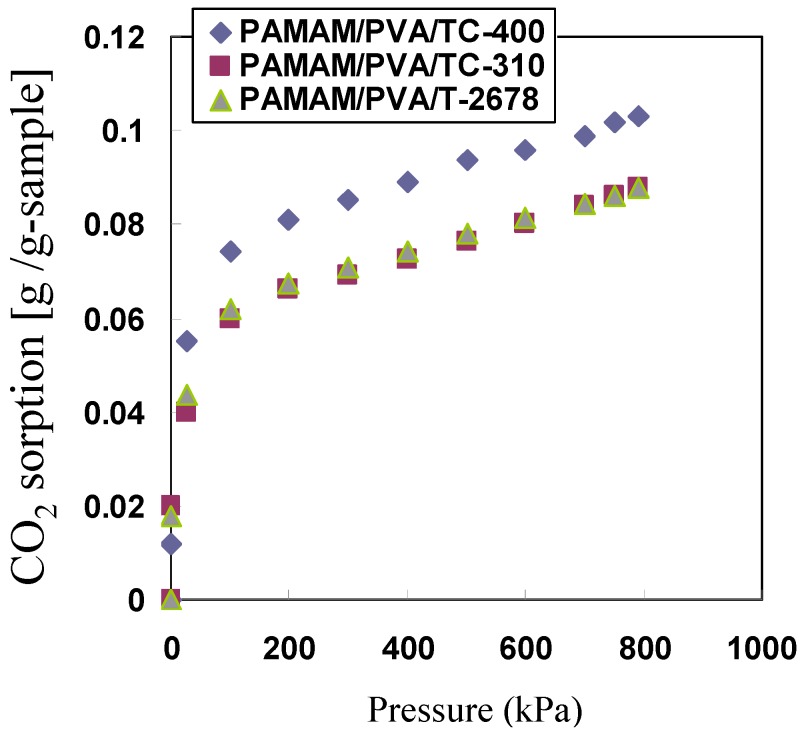
Effect of cross-linker on CO_2_ sorption properties of the PAMAM/cross-linked PVA membranes at 40 °C.

To further investigate the effect of the ethanol amino groups on facilitating CO_2 _sorption, H_2_O and CO_2_ sorption onto the membranes were examined and the results are shown in Figure 6 and Figure 7. The PAMAM/PVA/TC-400 hybrid membrane showed higher H_2_O and CO_2_ sorption when compared with the hybrid membranes prepared with the other two Ti cross-linkers. Consequently, the PAMAM/PVA/TC-400 hybrid membrane showed higher CO_2_ permeance and selectivity.

### 3.4. Effect of CO_2_ Partial Pressure on CO_2_ Separation Properties

**Figure 8:**
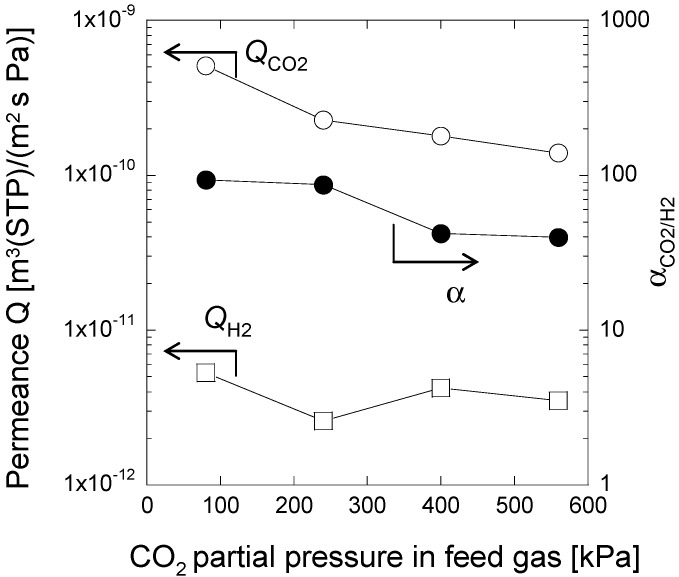
Effect of CO_2_ partial pressure in the feed gas on the CO_2_ separation properties of the PAMAM/cross-linked PVA composite membrane at 90% RH and 40 °C.

The effect of CO_2_ partial pressures on CO_2_ separation properties using the composite membrane of smaller thickness prepared by casting precursor solution of PAMAM/PVA/TC-400 onto a PVDF support membrane is shown in Figure 8. In general, as observed, CO_2_ permeance decreased with increasing CO_2_ partial pressures in the feed gas. Conversely, H_2_ permeance was relatively constant and only decreased slightly as CO_2_ partial pressure increased. As a result, the membrane showed high *Q*_CO2_ (1.1 × 10^−1^^0^ m^3^ (STP)/(m^2^·s·Pa)) and α_CO2/H2_ (39) even at a high CO_2_ partial pressure (*i.e*., 560 kPa).

A decrease in the permeance of CO_2_ as pressure increases is typically observed when gas separation takes place via an improved transport mechanism process [14,16], as indicated by the CO_2_ sorption behaviors. CO_2_ sorption values increased from 0 to 0.075 g/g-membrane with increasing CO_2_ partial pressures from 0 to 100 kPa, followed by a further increase from 0.075 to 0.094 g/g-membrane with increasing CO_2_ partial pressures from 100 to 600 kPa. CO_2_ sorption was facilitated by interactions between PAMAM dendrimer and CO_2_ molecules. CO_2_ sorption rates decreased as pressure increased. Moreover, CO_2_ sorption sites in the membrane do not increase. As mentioned above, permeance of CO_2_ decreased with increasing gas partial pressures. Conversely, H_2_ sorption was hindered by the PAMAM dendrimers because of the absence of CO_2_ chemical affinities. H_2_ transport in the membrane was independent of the gas pressure and H_2_ permeance remained unchanged.

## 4. Conclusions

PAMAM was successfully immobilized in a cross-linked PVA matrix using three types of Ti cross-linkers to form PAMAM/cross-linked PVA hybrid membranes. The nanoindentation and TG analyses demonstrated that cross-linking was effective to improve the mechanical strength and thermal stability of the PAMAM/PVA hybrid membrane. CO_2_ permeance and selectivity were different depending on the type of Ti cross-linker. PAMAM/PVA/TC-400 hybrid membrane showed higher H_2_O and CO_2_ sorption than other hybrid membranes prepared with the other Ti cross-linkers, and the highest CO_2_ separation capabilities. Moreover, the PAMAM/PVA/TC-400 hybrid membrane (thickness: 1.5 µm) showed CO_2_/H_2 _selectivity over 30 and CO_2_ permeance over 1.0 × 10^−1^^0^ m^3^ (STP)/(m^2^·s·Pa) at 560 kPa CO_2_ partial pressure. The PAMAM dendrimer/cross-linked PVA membrane shows great potential for CO_2_ separation from H_2_ in high-pressure applications such as the IGCC process.
